# Improving patient understanding and outcomes in myelodysplastic syndromes - An animated patient guide to MDS with visual formats of learning

**DOI:** 10.1016/j.lrr.2022.100328

**Published:** 2022-05-25

**Authors:** David A. Sallman, Rafael Bejar, Guillermo Montalban-Bravo, Sandra E. Kurtin, Alan F. List, Guillermo Garcia-Manero, Stephen D. Nimer, Casey L. O'Connell, Dale Schaar, Janice Butchko, Tracey Iraca, Stephanie Searle

**Affiliations:** a Department of Malignant Hematology, Moffitt Cancer Center Tampa, FL, United States; b Division of Hematology and Oncology, MDS Center of Excellence, University of California, San Diego La Jolla, CA, United States; c Department of Leukemia, University of Texas MD Anderson Cancer Center Houston, TX, United States; d University of Arizona Cancer Center, University of Arizona Tucson, AZ, United States; e Precision BioSciences Durham, NC, United States; f Department of Medicine, Biochemistry & Molecular Biology, University of Miami Miller School of Medicine, Sylvester Comprehensive Cancer Center Miami, FL, United States; g Jane Anne Nohl Division of Hematology, University of Southern California Los Angeles, CA, United States; h Rutgers Cancer Institute of New Jersey New Brunswick, NJ, United States; i MDS Foundation, NJ, United States; j Mechanisms in Medicine Inc., Toronto, ON, Canada

**Keywords:** Health literacy, Health outcomes, Myelodysplastic syndromes, Patient education, Visual formats of learning

## Abstract

**Objectives:**

Patient education resources that address barriers to health literacy to improve understanding and outcomes in myelodysplastic syndromes (MDS) are limited. The aim of this study was to evaluate the impact and outcomes benefits of *An Animated Patient's Guide to Myelodysplastic Syndromes (MDS)* cancer educational modules (which includes the ‘You and MDS’ website and YouTube hosted resources) related to MDS education, awareness, understanding and health outcomes.

**Methods:**

This was a retrospective study of learner feedback, metrics, and utilization data from July 2018 to August 2021. We evaluated audience reach (number of visit sessions, unique visitors, page views) and calculated top views by media type (animation, expert video, patient video, and slide show) and top retention videos from the modules. We also assessed the educational impact and utilization through learner feedback surveys.

**Results:**

During the study period, ‘You and MDS’ had 233,743 views worldwide of which 104,214 were unique visitors and 78,161 (or 76% unique visitors) were from the United States. Of these, 61% were patients; 29% family members or caregivers; 5% were healthcare providers and 5% represented other groups. Most popular topics viewed among the animations were “Understanding Myelodysplastic Syndromes (MDS)” (40,219 views), “Managing and Treating MDS” (19,240 views), “Understanding Erythropoiesis” (17,564 views.) The most popular expert videos viewed were “What is iron overload, and how it is treated?” (20,310 views), “How serious a cancer is MDS? What is the prognosis for MDS?” (8,327 views), “What is MDS?” (3,157 views). Of participants who completed the online feedback survey, ≥ 95% reported improved knowledge gains and commitments to change.

**Conclusions:**

MDS patients using ‘You and MDS - An Animated Patient's Guide to MDS’ and its visual formats of learning represented a wide U.S. and global learner audience. This MDS educational resource had a significant impact on improved understanding among patients, families, and caregivers. Continued efforts should be made to provide patient-effective resources that address health literacy, improve patient understanding, and address educational needs that respond to the concerns of patients to achieve better quality of life and improved health outcomes in MDS.

Myelodysplastic syndromes (MDS) represent a heterogeneous group of hematologic malignancies that are not well understood, and are characterized by ineffective hematopoiesis, variable cytopenias, and risk of progression to acute myeloid leukemia [1]. MDS is a significant cause of cancer morbidity and mortality in the United States, with an estimated 10,000 new diagnoses each year [2]. The prevalence of MDS is estimated to be between 60,000 – 170,000 patients in the US [3] The Revised International Prognostic Symptom Score (IPSS-R) median survival for low-risk patients may extend to 8.8 years; however, in high-risk patients the median survival may be a mere 0.8 years [4]. Advanced age is the predominate risk factor, with a median age of diagnosis of 71-76 years [5].

As survival rates for MDS patients are poor, understanding mechanisms of early events in disease development and new approaches for early detection and management are necessary for better outcomes [6]. In an Internet-based survey among 358 patients with MDS, 55% of patients did not know their IPSS score; 42% were unaware of their blast percentage; 28% were unaware of their cytogenetics and 16% of patients felt treatment would be curative [7]. Patients with high-risk disease were more likely to think their therapy would be curative than those with lower-risk disease. Patients with MDS have a limited understanding of their disease characteristics, prognosis, and treatment goals. Little information is available regarding how aware MDS patients are of their disease severity, prognosis, and treatment outcomes [8].

One of the key factors that can negatively impact patients' health outcomes is low health literacy [[9], [10], [11], [12], [13]]. Prior studies have linked lower levels of health literacy to poorer health-related quality of life [14] and a lower likelihood of receiving chemotherapy (cancer patients) [15].

In our study, we evaluated the impact of Myelodysplastic Syndromes Foundation's (MDSF's) ‘*You and MDS: An Animated Patient's Guide to MDS’* education modules using visual formats of learning to improve patient understanding and address educational needs over a period of 3 years [[16], [17]]. We monitored an audience of patients, families, and caregivers to determine audience learning activities, feedback, and interactions. We assessed the efficacy of *‘You and MDS’* and its role in addressing knowledge gaps and understanding by reducing learning barriers for patients to make informed decisions and partner with their health care providers to attain optimal health outcomes.

## 1 Materials and methods

### 1.1 Study design and participants

This is a retrospective study of the *‘You and MDS’* website and YouTube audience metrics for learner activities related to MDS patient education and allied learner lay audiences, conducted from July 2018 through August 2021; a period of 38 months. With the *‘You and MDS’* website launch, we utilized a series of outreach efforts using social media communications (YouTube, Facebook, Twitter, Instagram) to address awareness and access which included MDS Foundation's existing patient and family audiences, and *de novo* audiences in the United States and globally. Future efforts will continue expansionary opportunities to use social media assets to promote this resource to a wide audience of learners. Although participants visiting the *‘You and MDS’* modules on the website and YouTube channels are largely comprised of patients, and their families and caregivers, a significant number of users of the English-language resource are health care professionals validating the benefits of this resource in MDS patient clinics and care centers.

### 1.2 Content development and access

**Fig. 1:**
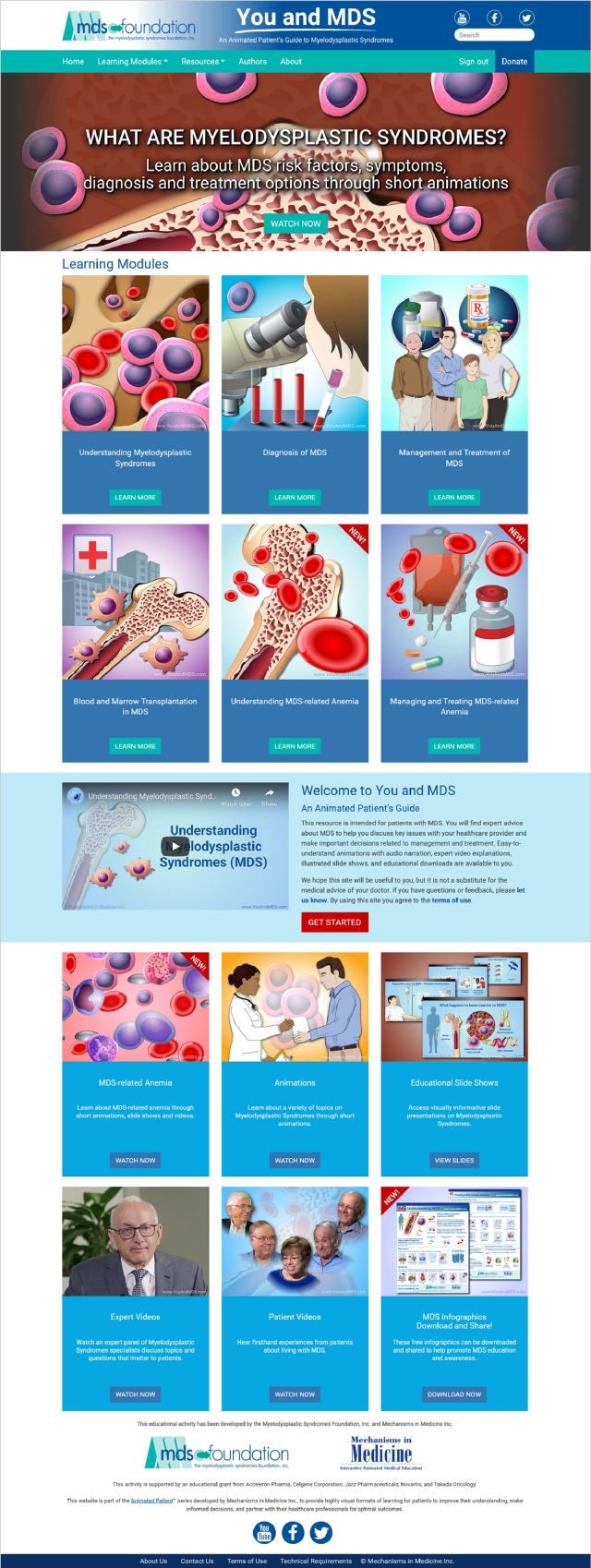
*You and MDS* website (https://www.YouandMDS.com).

**Fig. 2:**
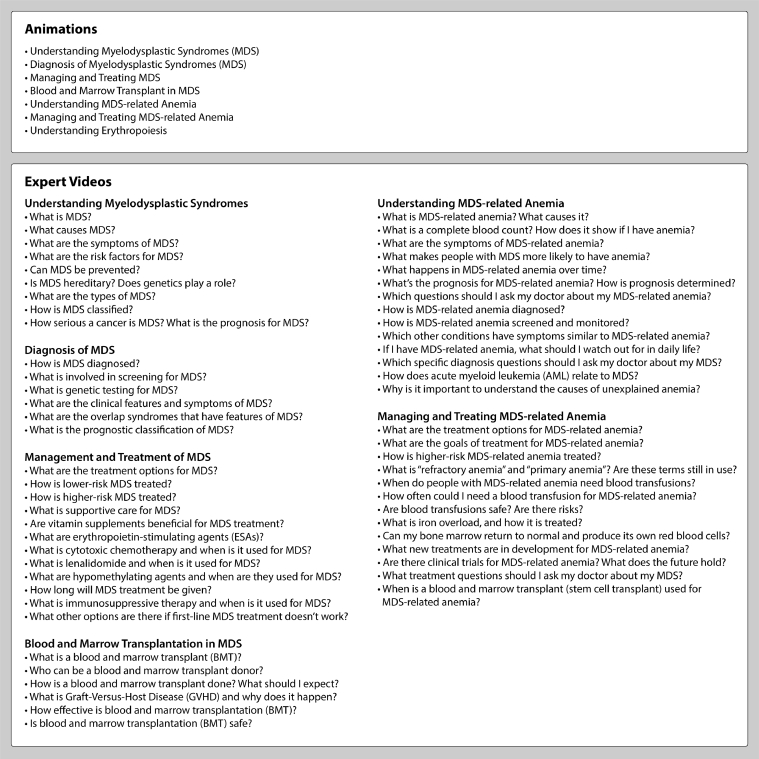
Overview of educational Animations and Expert Video content from *You and MDS* program.

The educational content for the *‘You and MDS’* resources were developed by MDS Foundation's multidisciplinary scientific advisors consisting of hematologists, oncologists, stem cell transplant specialists, nurse practitioners and health care providers at MDS Centers of Excellence. Online content is easily accessible, highly visual in nature, and interactive in presentation (Fig. 1). The education materials were designed to serve a lay audience with a grade 6 to 8 health literacy level (as assessed by literacy online evaluation tools). Each module was created to be succinct, practical, informative, evidence-based, patient-centric and aligned with the chosen learning objectives. The MDS educational curriculum is comprehensive in content, with opportunities to expand learning content in the future (Fig. 2).

The ‘You and MDS’ content contains animations, expert videos, and patient experience videos, slide shows and infographics. Each of the animations and videos were developed to be viewed within a 3 to 7 minutes timeframe. The MDS education modules can be accessed directly from the MDS Foundation's website at www.https://www.mds-foundation.org or directly through an online resource, www.youandmds.com or a YouTube channel at https://www.youtube.com/c/YouandMDS/featured. The list of accessible animations, expert videos, slide shows, infographics, and patient experience videos is illustrated in Tables 1 and 2. The website provides learner interactivity; a learner ‘pop-quiz’ allows users to self-test their knowledge skills and a homepage invitation prompts users to provide comments via an optional feedback survey.

**Table 1:** Top 5 Animations and Top 10 Expert Videos by Frequency.

Video Type	Video Title	YouTube	APP	Total
Animations	Understanding Myelodysplastic Syndromes (MDS)	21,887	18,332	40,219
	Managing and Treating MDS	11,243	7,997	19,240
	Understanding Erythropoiesis	16,526	1,038	17,564
	Diagnosis of Myelodysplastic Syndromes (MDS)	4,551	6,136	10,687
	Blood and Marrow Transplantation in MDS	3,468	3,436	6,904
Expert videos	What is iron overload, and how it is treated?	20,228	82	20,310
	How serious a cancer is MDS? What is the prognosis for MDS?	5,630	2697	8,327
	What is MDS?	187	2970	3,157
	What are the symptoms of MDS?	597	1723	2,320
	What causes MDS?	287	1841	2,128
	Are vitamin supplements beneficial for MDS treatment?	370	1521	1,891
	What are the types of MDS?	358	1204	1,562
	What are erythropoietin-stimulating agents (ESAs)?	970	571	1,541
	What are the risk factors for MDS?	93	1245	1,338
	How is lower risk MDS treated?	160	1079	1,239

**Table 2:** Top 5 Animations and Top 10 Expert Videos by Highest Retention Viewed.

Video Type	Video Title	Average Percentage Viewed
Animations	Managing and Treating MDS	58.9
	Understanding Myelodysplastic Syndromes (MDS)	56.7
	Diagnosis of Myelodysplastic Syndromes (MDS)	56.3
	Understanding MDS-related Anemia	54.5
	Understanding Erythropoiesis	53.9
Expert videos	What are the treatment options for MDS?	83.5
	What are the risk factors for MDS?	82.9
	Are vitamin supplements beneficial for MDS treatment?	82.6
	Which other conditions have symptoms similar to MDS-related anemia?	81.7
	How is MDS diagnosed?	80.9
	What is involved in screening for MDS?	80.9
	Can my bone marrow return to normal and produce its own red blood cells?	80.8
	What new treatments are in development for MDS-related anemia?	80.5
	What are the symptoms of MDS-related anemia?	80.3
	How is MDS-related anemia screened and monitored?	80.1

Retention determined by the percentage of the total video length viewed.

### 1.3 User metrics measurement and statistical analysis

Data were reported as frequencies, and proportions and mean (±SD), where appropriate. We evaluated audience reach, demographics, and metrics such as the number of visit sessions, number of unique visitors, page views, duration of page views, and duration of video views for the *‘You and MDS’* website and the *‘You and MDS’* YouTube channel. We also calculated top views, top views by media type (animation, expert video, patient experience video, storyboard slide show, infographic) and top retention videos overall for the website and YouTube channel. Finally, we assessed the educational impact of the *‘You and MDS”* program from the metrics data; the user utilization of learning resources; accessing data of areas of high and low interest; and monitoring and collecting specific learner feedback survey data.

## 2 Results

### 2.1 Participant characteristics

During the study period, the *‘You and MDS’* website (Fig. 1) and YouTube channel had 233,743 total views (‘*You and MDS’* website = 134,863; YouTube = 98,880). Overall, the educational content was accessed by 104,214 unique visitors from 141 countries. Most of the unique visitors to the website (76% or 79,203) were from the United States while 25,038 unique visitors were from other countries; namely United Kingdom (4%); Canada (4%); China (3%); Australia (2%) and other countries (11%).

Of the 1,850 respondents who provided feedback, more than half of participant responses (61%) identified as MDS patients; the remainder were family or caregivers (29%), health care providers (5%), and other (5%).

### 2.2 Animation, expert videos, and patient videos

Table 1 lists the most popular topics for the animation and videos. “Understanding Myelodysplastic Syndromes (MDS)” (40,219 views), “Managing and Treating MDS” (19,240 views), “Understanding Erythropoiesis” (17,564 views), “Diagnosis of Myelodysplastic Syndromes (MDS)” (10,687 views), “Blood and Marrow Transplantation in MDS” (6,904 views) were the top animations viewed, respectively.

The most popular expert videos viewed by the participants were “What is iron overload, and how it is treated?” (20,310 views), “How serious a cancer is MDS? What is the prognosis for MDS?” (8,327 views), “What is MDS?” (3,157 views), “What are the symptoms of MDS?” (2,320 views), and “What causes MDS?” (2,128 views) (Table 1).

### 2.3 Viewer retention for animations and videos

Table 2 lists the top 5 animations and top 10 videos by viewer retention (as determined by the percentage of the total video length viewed). On average, 70.1% of each video's content was viewed.

### 2.4 Direct comments from patients, caregivers and visitors

Table 3 lists a sample of direct quotes of patients, caregivers and website visitors showing different or similar perspectives on the educational information received. This feedback was based on “Short Survey Question # 4. Do you have any other comments or unanswered questions?” and “Long Survey Question # 12. Other questions I have about MDS?”

**Table 3:** Direct Comments from Patients, Caregivers and Website Visitors in Response to the Educational Information Received.

Visitor Type	Direct Comment
Patient	I had two stem cell transplants - very good and clear explanation of MDS.
Patient	I was diagnosed 24 years ago, have been treated weekly for 15 years; and I was a PhD Organic/Biochemist specialist in chemical mechanisms in living systems. I enjoyed this, though, since it is a good simple explanation, I can send to people who ask about it.
Patient	Info provided enhances and validates what my hematologist has discussed with me. Appreciate the specific info re treatments.
Patient	Thank you. This information has been very helpful. My blood counts have been decreasing for the past several years and have leveled off. I am now on a 12-month check. Pre-MDS. Have an excellent doctor and team.
Patient	This was very informative as I was just diagnosed within the week.
Patient	Very grateful for the video and all the information provided. I have learned a lot more here than at the Dr's office.
Patient	My hematologist considers me to be Very Low to Low risk. This week I have started Decitabine treatments for anemia because my HBG is below 7.0. My hematologist has been very informative and has informed me exactly what your animation has shown. I have forwarded this animation to my siblings and family so that they will have a better understanding of my MDS.
Patient	Will pass this on to my family and nurse.
Patient	I can't find the list of questions to ask my doctor.
Patient	Really great videos. Thank you! Especially the animated ones at the top. And the patient perspectives helpful.
Patient	The above questions are not relevant, only because I already talked to my physician as needed.
Patient	I have not been able to find much about how one physically feels with MDS... besides blood tests and level of tiredness what are the symptoms to look for?
Other	Excellent videos.
Other	Excellent, informative, easy to understand!
Other	Very informative website.
Other	Thank you, this site is very helpful.
Caregiver	Thank you for making these videos. My mom was just diagnosed with the del(5q) version.
Caregiver	Thank you for the videos. Very helpful! I think this will also help my 92-year-old mother.
Caregiver	Stem cell transplant is scheduled…. So far, it seems my husband's MDS is right on track with what I have seen on this website.

### 2.5 Participant knowledge and commitment to change

**Fig. 3:**
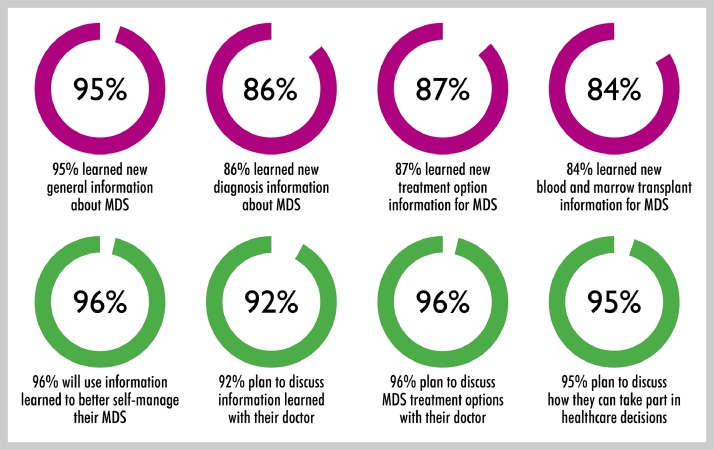
Patients who experienced improved outcomes.

Among the 1,850 participants who completed the online feedback survey, approximately 95% reported that they had learned new MDS information. 86% learned new diagnosis information, 87% learned new treatment options, and 84% learned new blood and marrow transplant information for MDS. Most participants (96%) expressed a commitment to change, in terms of using the information to better manage their MDS, and indicated their intention to engage with their doctor in discussions (Fig. 3).

## 3 Discussion

During the 3-year study period, the MDS Foundation's ‘You and MDS – An Animated Patient's Guide to MDS’ education modules garnered 233,743 views and approximately 104,214 unique participants. The most popular topic among the animations was “Understanding Myelodysplastic Syndromes (MDS)” and the most popular expert video viewed was “What is iron overload, and how it is treated?”. Participants who completed the online feedback survey reported knowledge gains and commitment to change by engaging with their physician or implementing a newly learned self-management action.

Myelodysplastic syndromes cause substantial morbidity, mortality, and costs. While the efforts to improve MDS outcomes are being pursued in areas related to early diagnosis, surveillance, and advances in treatments, reliable educational resources specific to MDS patient needs and understanding of disease are limited. Current cancer patient education resources and content is predominantly text based [[18], [19], [20], [21], [22], [23], [24], [25], [26]] and most often written at literacy levels above the comprehension level of lay persons, even though National Institutes of Health and the American Medical Association recommend a 5th grade to 8th grade target comprehension level for patient education materials [[27], [28]].

The Myelodysplastic Syndromes Foundation's ‘You and MDS - An *Animated Patient's Guide to MDS’* uses visual formats of learning to address patient barriers to health literacy and provides accessible, easy-to-follow, evidence-based resources. Multidisciplinary expert author faculty guide evidence-based content development of the website and You Tube resources. Educational content is simple to understand; brief animations with narration focusing on frequently asked questions and key disease concepts are easily understood. Short video segments of experts answering commonly asked questions complement learning tools; videos of patients sharing their MDS disease experiences and downloadable slide shows, and infographics add to the content covered by animations. The content of the education modules aligns with the health literacy literature on the effectiveness of visual aids and video formats in patient education [29]. The feedback survey on self-reported gains in learning, competence, and intention to change also align with Moore and colleagues [30], which is consistent with level 4 outcomes for continuing medical education. This unique format of learning permits feedback from learners through voluntary self-evaluation tools and encourages learners to indicate benefits in knowledge acquisition; learners indicate their intent to discuss an intervention with their hematology/oncology provider and the interactive format elicits and reports on improved learner understanding. Education content helps learners make informed choices and share in decisions with their health care providers regarding MDS management.

Some of the strengths of our study assessed over a period of 3 years include wide reach of this program (US and global audience), use of a multidisciplinary expert faculty from MDS Centers of Excellence in the development of content, addressing health literacy impediments, and improving understanding related to MDS patient needs. Patients are capable of participating in shared decisions through educational modules, which promote patient satisfaction with their health care experience [[31], [32]]. Educational animations and content are continuously being added for the English and Spanish programs, while other language programs are feasible in the future to benefit global MDS communities.

The limitation of this study includes its retrospective design and a relatively small percentage of users (∼1.8%) who took part in the optional feedback survey. Our study is reliant on self-reported data, with no opportunity to independently confirm participants' reports of their disease history and treatment. Another limitation is the potential for learner response bias in the population who elected to complete online self-evaluation and feedback. Moreover, You Tube learner audiences are unlikely to provide feedback, without visiting the ‘You and MDS’ website. Although the ‘You and MDS’ resources received approximately 233,743 views worldwide, few (n = 1,850) participated in the feedback survey. This is because the feedback survey was optional (no monetary compensation is provided). Furthermore, additional requests for feedback would potentially deter voluntary responses and the goal was to facilitate a broad response. In the future an opportunity will be evaluated to compare the results from incentivized responses with results from the current audience of patients, families and caregivers utilizing this online resource. It is anticipated that introducing such an incentive will improve feedback and provide greater insights related to representation of learner knowledge translation and highlight unmet needs. Collection of learner response data will guide future content development and address learning improvements for MDS patients. The program also lacked a formal pre-survey/post-survey assessment. Because of the nature of the feedback survey, we were limited in gathering in-depth learner data, however future goals are to improve the quality of information from heterogeneous learner audiences.

In conclusion, *An Animated Patient's Guide to Myelodysplastic Syndromes: You and MDS,* that uses visual formats of learning, demonstrates wide reach, and has vast potential to improve understanding and benefit health outcomes by informing MDS patients, families, and caregivers.

Future areas of focus for this MDS patient resource will be based directly on feedback from patients and families in order to provide education on new therapies, clinical trials, diagnostic tools, and developments in MDS transplant interventions and ultimately improve patient outcomes. Continued efforts should be made to provide patient resources that address health literacy barriers, increase disease understanding, improve health outcomes and quality of life.

## Declaration of Competing Interest

No conflict of interest in relation to this manuscript. No funding was requested or received for the development of this manuscript. *You and MDS: An Animated Patient's Guide to Myelodysplastic Syndromes* is supported by unrestricted education grants from Acceleron Pharma, Bristol-Myers Squibb, Celgene Corporation, Jazz Pharmaceuticals, Novartis, and Takeda Oncology. Mechanisms in Medicine, the developers of this resource, declare no conflict of interest in the development of the program.

## References

[1] Nimer S.D. (2008). Myelodysplastic syndromes. Blood.

[2] Myelodysplastic syndromes. MDS statistics. https://www.cancer.net/cancer-types/myelodysplastic-syndromes-mds/statistics.

[3] Leukemia and Lymphoma Society. https://www.lls.org/research/myelodysplastic-syndrome-mds-research-funded-lls.

[4] American Cancer Society. Survival statistics for myelodysplastic syndromes. https://www.cancer.org/cancer/myelodysplastic-syndrome/detection-diagnosis-staging/survival.html.

[5] Leukemia and Lymphoma Society. https://www.lls.org/research/myelodysplastic-syndrome-mds-research-funded-lls.

[6] Sekeres M.A., Maciejewski J.P., List A.F., Steensma D.P (2011). Perceptions of disease state, treatment outcomes, and prognosis among patients with myelodysplastic syndromes: results from an internet-based survey. The Oncologist.

[7] Sekeres M.A., Maciejewski J.P., List A.F., Steensma D.P (2011). Perceptions of disease state, treatment outcomes, and prognosis among patients with myelodysplastic syndromes: results from an internet-based survey. The Oncologist.

[8] Sekeres M.A., Maciejewski J.P., List A.F. et al. Perceptions of disease state, treatment outcomes, and prognosis among patients with myelodysplastic syndromes: results from an internet-based survey. 08 April 2011. 10.1634/theoncologist.2010-0199 PMC3228221 21478277

[9] Berkman ND, Sheridan SL, Donahue KE (2011). Low health literacy and health outcomes: an updated systematic review. Ann Intern Med.

[10] Levy H, Janke A. (2016). Health literacy and access to care. J Health Commun.

[11] Hersh L, Salzman B, Snyderman D. Health literacy in primary care practice. Am Fam Physician. 2015; 92:118–124. 26176370

[12] Weiss BD. (2007). Manual for Clinicians.

[13] Nielsen-Bohlman L, Panzer AM, Kindig DA, Institute of Medicine (US) Committee on Health Literacy (2004). Health Literacy: A Prescription to End Confusion.

[14] Halverson JL, Martinez-Donate AP, Palta M (2015). Health literacy and health-related quality of life among a population-based sample of cancer patients. J Health Commun.

[15] Busch EL, Martin C, DeWalt DA (2015). Functional health literacy, chemotherapy decisions, and outcomes among a colorectal cancer cohort. Cancer Control.

[16] The Myelodysplastic Syndromes Foundation [website home page]. 2021. Available at: https://www.mds-foundation.org/.

[17] Animated Patients Guide to MDS [website home page]. 2021. Available at www.YouAndMDS.com.

[18] NCCN Guidelines for Patients (2021). National Comprehensive Cancer Network.

[19] Storino A, Castillo-Angeles M, Watkins AA (2016). Assessing the accuracy and readability of online health information for patients with pancreatic cancer. JAMA Surg.

[20] Prabhu AV, Hansberry DR, Agarwal N (2016). Radiation oncology and online patient education materials: deviating from NIH and AMA recommendations. Int J Radiat Oncol Biol Phys.

[21] Prabhu AV, Donovan AL, Crihalmeanu T (2018). Radiology online patient education materials provided by major university hospitals: do they conform to NIH and AMA guidelines?. Curr Probl Diagn Radiol.

[22] Prabhu AV, Crihalmeanu T, Hansberry DR (2017). Online palliative care and oncology patient education resources through Google: do they meet national health literacy recommendations?. Pract Radiat Oncol.

[23] Hansberry DR, Agarwal N, John ES (2017). Evaluation of internet-based patient education materials from internal medicine subspecialty organizations: will patients understand them?. Intern Emerg Med.

[24] Weiss KD, Vargas CR (2016). Readability analysis of online resources related to lung cancer. J Surg Res.

[25] Hansberry DR, Patel SR, Agarwal P (2017). A quantitative readability analysis of patient education resources from gastroenterology society websites. Int J Colorectal Dis.

[26] Prabhu AV, Kim C, Crihalmeanu T (2017). An online readability analysis of pathology-related patient education articles: an opportunity for pathologists to educate patients. Hum Pathol.

[27] Weiss BD. (2007).

[28] NIH. Clear Communication. Clear and Simple. Available: https://www.nih.gov/institutes-nih/nih-office-director/office-communications-public-liaison/clear-communication/clear-simple. 2007.

[29] Nienkamp M. Visual learning tools overcome health literacy. [PSQH e-Newsletter]. July–August 2006. Available at: https://www.psqh.com/julaug06/visual.html . Accessed February 1, 2021.

[30] Moore Jr DE, Green JS, Gallis HA (2009). Achieving desired results and improved outcomes: integrating planning and assessment throughout learning activities. J Contin Educ Health Prof.

[31] Kane HL, Halpern MT, Squiers LB (2014). Implementing and evaluating shared decision making in oncology practice. CA Cancer J Clin.

[32] Katz SJ, Hawley S. (2013). The value of sharing treatment decision making with patients: expecting too much?. JAMA.

